# Anemia prevalence and treatment patterns in chronic kidney disease: a nationwide observational study using a Japanese primary care database (2018–2023)

**DOI:** 10.1007/s10157-026-02873-2

**Published:** 2026-06-30

**Authors:** Tetsuhiro Tanaka, Shinji Asada, Kohei Suzuki, Yuki Toyoshima

**Affiliations:** 1 Department of Nephrology, Tohoku University Graduate School of Medicine, Miyagi, Japan; 2 Medical Affairs Department, Kyowa Kirin Co., Ltd., Tokyo, Japan

**Keywords:** Anemia, Chronic kidney disease, Primary care, Database

## Abstract

**Background:**

Anemia in chronic kidney disease (CKD) is associated with poor outcomes, including CKD progression, mortality, and cardiovascular events. Standard treatments include iron supplements and injectable erythropoiesis-stimulating agents (ESAs). Oral hypoxia-inducible factor–prolyl hydroxylase inhibitors (HIF–PHIs) have recently emerged as a new treatment option, providing easier accessibility for general practitioners.

**Methods:**

This real-world descriptive study used the Japan Medical Data Survey database to analyze the prevalence of anemia, anemia treatment, and treatment type in non-dialysis patients with CKD and treated in primary care clinics. Data from 1 January 2018 to 31 December 2023 were assessed and analyzed by subgroups (year, CKD stage, patient age, treatment setting, sex, and region).

**Results:**

This study included 397,419 patients. Anemia treatment rates increased from 16.4% (2018) to 22.7% (2023), accompanied by an increased proportion of patients with hemoglobin levels ≥ 11 g/dL (78.8%–87.9%). From 2020, use of HIF–PHIs increased and use of ESAs decreased, with HIF–PHIs overtaking ESAs in 2022–2023. During 2022–2023 (N = 340,398), patients with more severe CKD stages had lower hemoglobin levels and an increased prevalence of anemia (11.6% [CKD G3a]–73.6% [CKD G5]). Older patients tended to have lower hemoglobin values, increased anemia prevalence, more severe CKD, and increased use of HIF–PHIs and ESAs.

**Conclusion:**

Use of HIF–PHIs in primary care settings in Japan is increasing, resulting in changes to anemia treatment received by patients with CKD.

**Supplementary Information:**

The online version contains supplementary material available at 10.1007/s10157-026-02873-2.

## Introduction

Anemia is a condition in which the oxygen-carrying capacity of a patient’s blood is insufficient to meet their requirements [1]. In patients with chronic kidney disease (CKD), the incidence of anemia is high [2] and is associated with increased mortality, hospitalization, serious cardiovascular events, and CKD progression [2, 3].

Anemia in CKD can be caused by low erythropoietin [4] and is treated with iron supplements and/or erythropoiesis-stimulating agents (ESAs). Treatment with ESAs for anemia in CKD has often been performed by nephrologists [5]. However, a 2014 Japanese nationwide database study showed that the proportion of patients with CKD receiving ESAs for anemia was lower in Japan than in the US, suggesting that some patients, particularly older Japanese patients, do not receive appropriate treatment [6, 7]. Another study at a single Japanese university hospital reported that non-nephrologists prescribed ESAs at far lower rates than nephrologists [8]. Because ESAs require injection and refrigeration, they can be difficult for non-nephrologist physicians to use in routine practice. These barriers may have contributed to the underuse of ESA therapy and suboptimal anemia management in CKD.

To address this, hypoxia-inducible factor–prolyl hydroxylase inhibitors (HIF–PHIs) were developed as oral treatments that can be stored at room temperature. Approval of HIF–PHIs for non-dialysis patients was granted in 2020 in Japan, and their use has increased through prescriptions by non-nephrologist physicians, particularly general practitioners (GPs), and through their inclusion in the Japanese Society of Nephrology (JSN) 2023 Chronic Kidney Disease Clinical Practice guidelines (JSN2023) [9].

CKD affects a large proportion of the Japanese population [10], with GPs playing an important role in CKD management in coordination with specialized departments. Patients with CKD are often elderly and tend to have multiple comorbidities, making clinical decision-making particularly complex in primary care. As GPs may have insufficient specialized knowledge and experience of treating anemia in CKD, such as the recommended hemoglobin management methods or intervention criteria, treatment with HIF–PHIs by GPs might result in suboptimal treatment of some patients. Therefore, there is a need to understand anemia treatment in CKD, particularly before and after the introduction of HIF–PHIs.

We used GP-based real-world data from the Japan Medical Data Survey (JAMDAS) database to analyze the prevalence of anemia, anemia treatment, and type of treatment (including ESAs and HIF–PHIs) in patients with CKD treated in primary care clinics, hypothesizing that the use of HIF–PHIs has increased by GPs. To understand treatment differences, including among groups previously reported to be undertreated (such as older patients [6]), we evaluated anemia treatment and type by patient subgroup and treatment setting.

## Materials and methods

### Study design and population

This was a real-world descriptive study using the JAMDAS database, an electronic health record database with data from approximately 5000 clinics and GPs in Japan (approximately 5% of all primary care clinics in Japan) [11].

The data extraction period was from 1 January 2017 to 31 December 2023. The entire cohort from 2018 to 2023 was analyzed, while the main analysis was limited to patients with index dates in 2022 and 2023 to focus on the period when HIF–PHIs were introduced. The observation start and end dates were the earliest and latest dates, respectively, in the patient’s medical record during the observation period (from 1 January 2018 to 31 December 2023). The index date (Day 0) was the date of the last hemoglobin measurement obtained during the observation period, except for patients with multiple hemoglobin measurements during the same year, for whom the date closest to 1 July was used.

Data were included for patients aged ≥ 18 years at the day of first observation, with one or more estimated glomerular filtration rate (eGFR) values of < 60 mL/min/1.73 m^2^ (per the Japanese eGFR estimation formula [12]), and with more than one serum hemoglobin measurement during the observation period. Data from 2017 were used to assess baseline characteristics, obtain covariate information, and apply exclusion criteria. Patients for whom an index date could not be set and patients for whom there were no medical records in the 180 days prior to the index date were excluded. Data from patients with a diagnosis of acute renal failure or a pregnancy record within 365 days of the index date, or a diagnosis of end-stage renal disease, malignancy, or eGFR < 5 mL/min/1.73 m^2^ prior to the index date, were also excluded.

This study was approved by the Kyowa Kirin Co., Ltd. Research Ethics Review committee (approval number: MA2024_005_0). As this study was conducted using anonymized data that were already available, it was considered outside the scope of the Ethical Guidelines for Medical and Biological Research Involving Human Subjects (Japanese Ministry of Health, Labour and Welfare).

### Measurements

Variables related to anemia and its management were evaluated. The codes used to identify diagnoses, medications, and tests are shown in Supplementary Table 1.

Anemia was considered present if the patient was diagnosed with anemia, had one or more hemoglobin measurements that met the criteria for anemia per the 2015 Japanese Society for Dialysis Therapy Guidelines for Renal Anemia in Chronic Kidney Disease (JSDT2015) [13] (defined as hemoglobin < 13.5 g/dL, < 12.0 g/dL, and < 11.0 g/dL for men aged < 60 years, 60–69 years, and ≥ 70 years, respectively, and < 11.5 g/dL and < 10.5 g/dL for women aged < 60 years and ≥ 60 years, respectively), was receiving one or more anemia medications, or had one or more blood erythropoietin measurements < 50 mIU/mL.

Hemoglobin categories included < 9 g/dL, 9– < 10 g/dL, 10– < 11 g/dL, 11– < 12 g/dL, 12– < 13 g/dL, and ≥ 13 g/dL.

Anemia medications included iron supplements (intravenous, oral, and iron-containing phosphate binders), ESAs (epoetin, darbepoetin alfa, and epoetin beta pegol), and HIF–PHIs (roxadustat, daprodustat, vadadustat, enarodustat, and molidustat sodium).

Anemia-related laboratory tests included measurements of hemoglobin, mean corpuscular volume (MCV), reticulocyte, ferritin, transferrin saturation (TSAT), iron, vitamin B12, folic acid, erythropoietin, and total iron binding capacity (TIBC).

CKD stage (G3a [eGFR ≥ 45 and < 60 mL/min/1.73 m^2^], G3b [≥ 30 and < 45 mL/min/1.73 m^2^], G4 [≥ 15 and < 30 mL/min/1.73 m^2^], or G5 [< 15 mL/min/1.73 m^2^]) and treatment setting (diabetes clinic, cardiology clinic, urology clinic, or home care; Supplementary Table 2) were also evaluated. If more than one eGFR value was available, the value from the day closest to the index date was used to determine the CKD stage.

### Statistical analysis

Continuous variables were summarized using means and medians with standard deviations, 95% confidence intervals, or interquartile ranges, as appropriate. Categorical variables were summarized by frequency and percentage. For anemia treatment rates, anemia treatment type, and anemia-related tests, percentages were calculated using patients with anemia as the denominator.

We conducted subgroup analyses of variables of interest (prevalence of anemia, hemoglobin levels, hemoglobin level categories, use and type of anemia medications, and implementation of anemia-related laboratory tests) by year, CKD stage, patient age, treatment setting, sex, and region. For cases with unknown date data, the day was considered “day 15”, but cases with unknown year or month data were assumed to be missing and were not imputed. Standardized coding and data extraction methods were used to minimize inconsistencies across facilities.

All analyses were performed using SAS 9.4 (SAS Institute Inc. 2023; Cary, NC, USA).

## Results

### Population

**Fig. 1:**
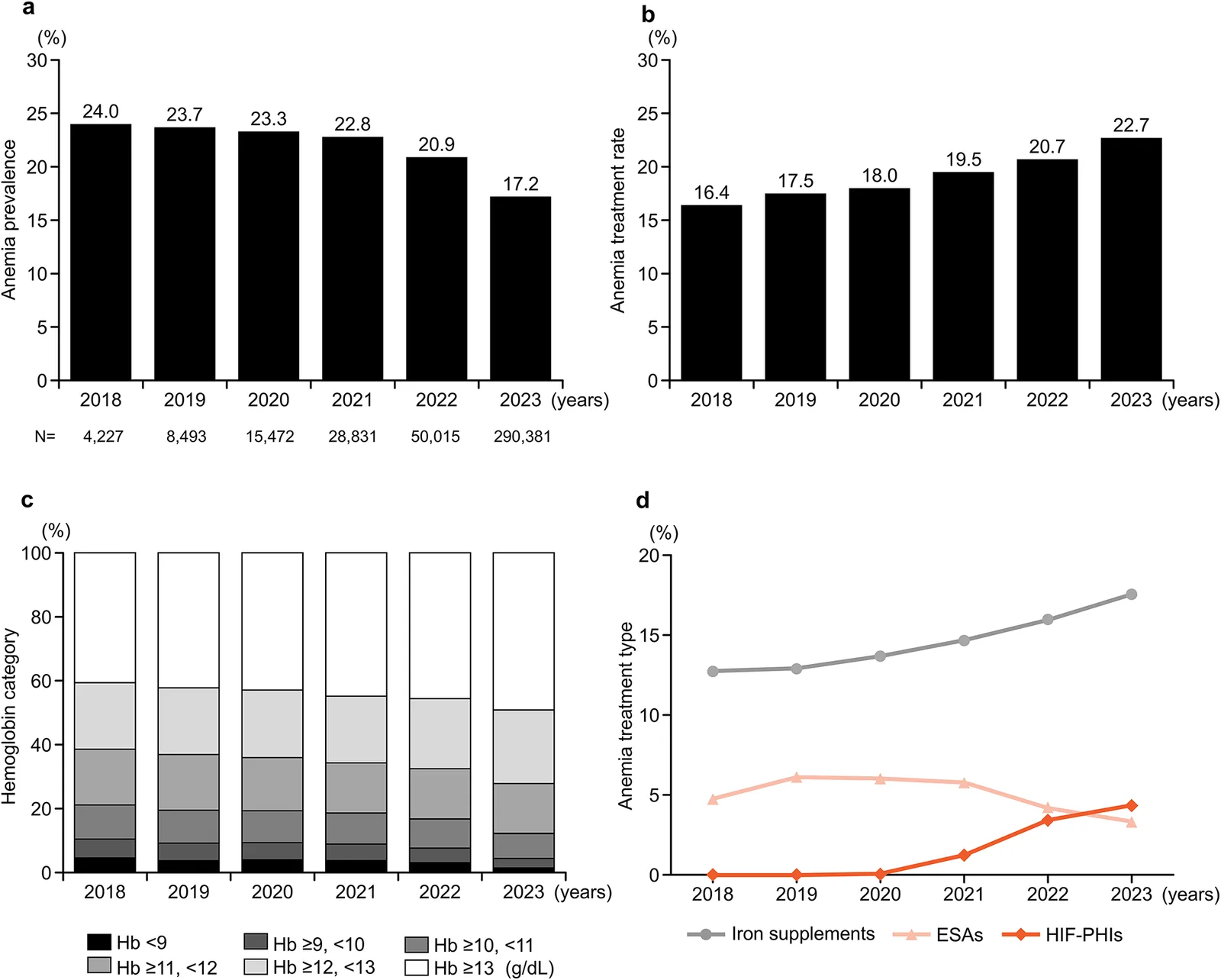
Anemia in patients with CKD between 2018 and 2023. **a** Anemia prevalence in patients with CKD over time. **b** Anemia treatment rate in patients with CKD and anemia. **c** Hemoglobin levels in patients with CKD and anemia. **d** Anemia treatment in patients with CKD and anemia. *CKD* Chronic kidney disease, *ESAs* erythropoiesis-stimulating agents, *Hb* Hemoglobin, *HIF–PHIs* Hypoxia-inducible factor–Prolyl hydroxylase inhibitors

The entire dataset composition (N = 397,419) is shown in Supplementary Fig. 1 with changes over time in anemia prevalence, treatment, hemoglobin levels, and anemia treatment from 2018 to 2023 shown in Fig. 1 and Supplementary Table 3.

Anemia treatment rates increased from 16.4% in 2018 to 20.7% in 2022 and 22.7% in 2023 (Fig. 1b). This increase was accompanied by a higher proportion of patients with improved hemoglobin levels, with the proportion of patients with levels ≥ 13 g/dL rising from 40.6% in 2018 to 45.6% in 2022 and 49.2% in 2023 (Fig. 1c). The proportion of patients with levels ≥ 11 g/dL increased from 78.8% in 2018 to 83.3% in 2022 and 87.9% in 2023. Use of ESAs decreased during 2022 and 2023, while use of HIF–PHIs increased from 2020 and overtook the use of ESAs during 2022 and 2023 (Fig. 1d). The use of iron supplements increased in line with the use of HIF–PHIs.

**Fig. 2:**
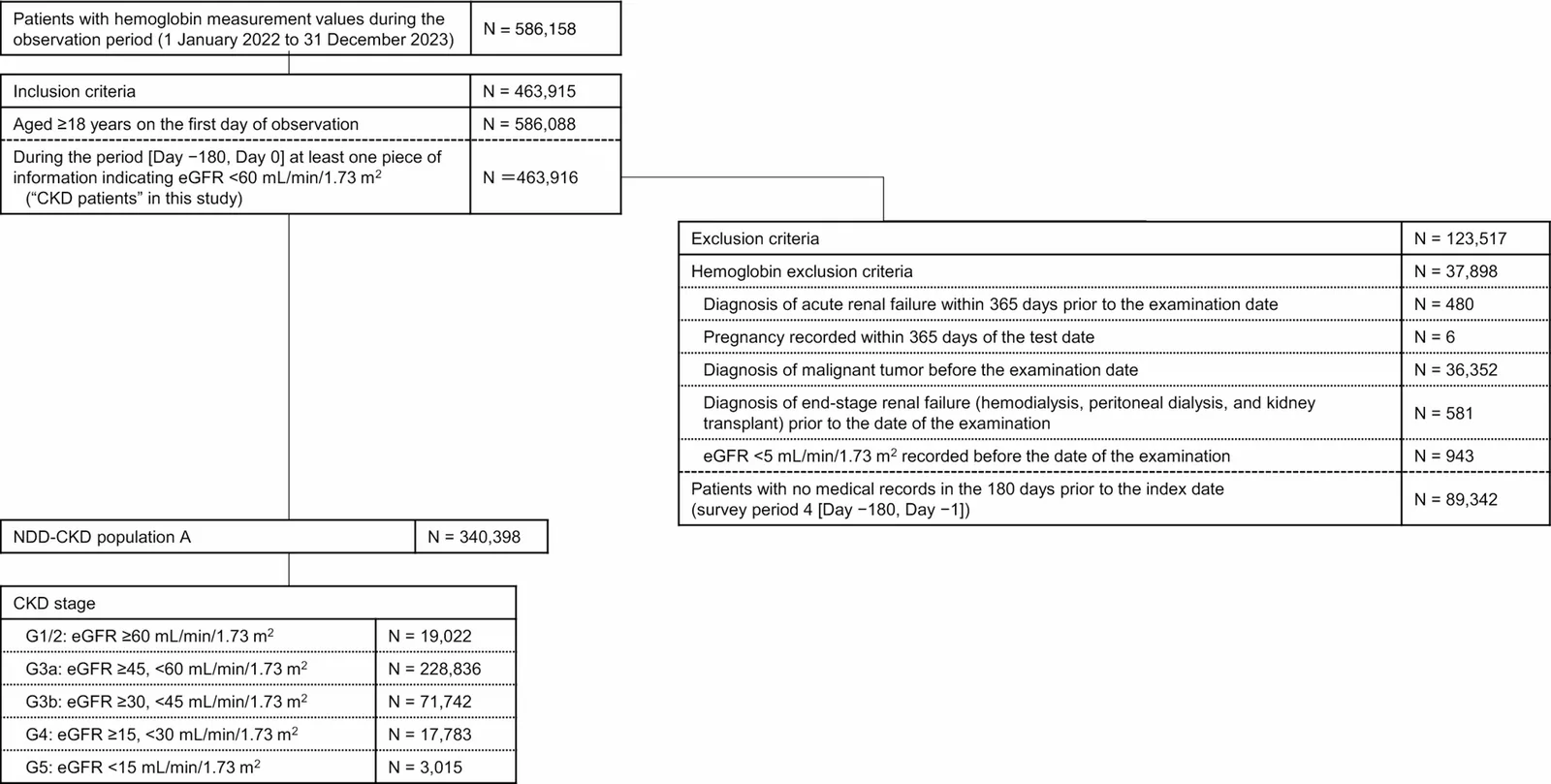
Dataset flow diagram between 2022 and 2023. *CKD* Chronic kidney disease, *eGFR* Estimated glomerular filtration rate, *NDD* Non-dialysis-dependent

The dataset composition (N = 340,398) for the main analysis period (2022–2023) is shown in Fig. 2. In the 2022–2023 dataset (Table 1 and Supplementary Table 4), 59.1% of the patients were female. Mean age was 76.24 years, the most common age category was 75–84 years (35.3%), and 83.9% of patients were aged ≥ 65 years.

**Table 1:** Patient characteristics between 2022 and 2023

		All patients (N = 340,398)
Sex (%)	Male	40.9
Age (years)	Mean ± SD	76.24 ± 11.63
Age category (%)	< 65	16.1
	65–74	24.5
	75–84	35.3
	85–94	21.7
	≥ 95	2.4
BMI (kg/m^2^)	Mean ± SD	23.50 ± 3.72
Comorbidity (%)	Hypertension	64.0
	Diabetes	32.1
	Dyslipidemia	52.4
	Heart failure	21.6
	Ischemic heart disease	14.6
	Stroke	13.3
	Peripheral arterial disease	1.5
Blood assessments		
eGFR (mL/min/1.73 m^2^)	Mean ± SD	49.34 ± 10.66
Serum albumin (g/dL)	Mean ± SD	4.01 ± 0.44
Uric acid (mg/dL)	Mean ± SD	5.69 ± 1.41
Sodium (mEq/L)	Mean ± SD	141.0 ± 2.9
Potassium (mEq/L)	Mean ± SD	4.28 ± 0.55
Chloride (mEq/L)	Mean ± SD	103.8 ± 3.4
Phosphate (mg/dL)	Mean ± SD	3.35 ± 0.60
Calcium (mg/dL)	Mean ± SD	9.12 ± 0.49
C-reactive protein (mg/dL)	Median (IQR)	0.110 (0.050, 0.370)
Urine assessments		
Urine protein (quantitative) (mg/dL)	Median (IQR)	11.0 (5.0, 32.0)
Urine protein (qualitative) (%)	Negative	5.4
	±	1.0
	1 +	0.7
	≥ 2 +	0.5
Urinary albumin (mg/dL)	Median (IQR)	17.7 (7.2, 57.5)

*BMI* body mass index, *eGFR* estimated glomerular filtration rate, *IQR* interquartile range, *SD* standard deviation

### Anemia prevalence 2022–2023

**Fig. 3:**
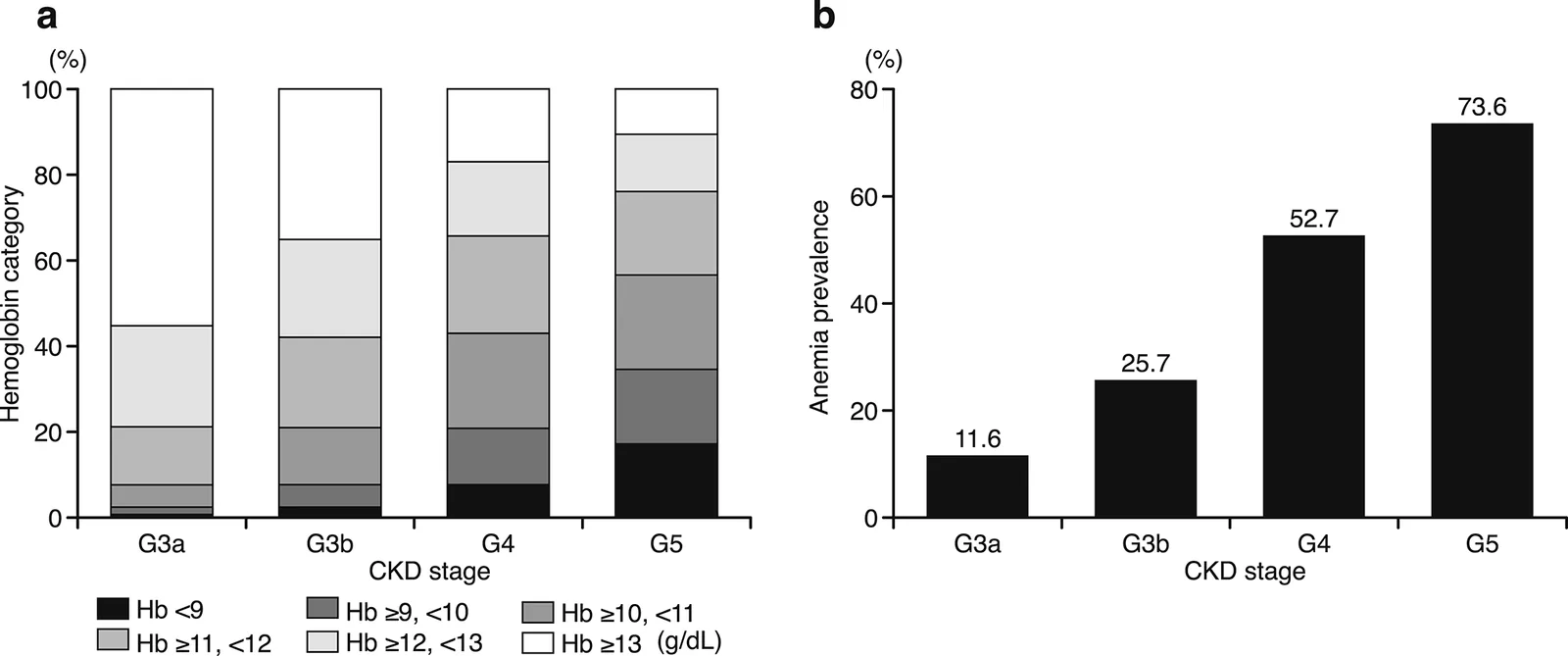
Hemoglobin levels and anemia prevalence by CKD stage between 2022 and 2023. **a** Hemoglobin levels by CKD stage. **b** Anemia prevalence by CKD stage. *CKD* Chronic kidney disease, *G* Grade, *Hb* Hemoglobin

The highest proportion of patients with hemoglobin values above the treatment target (≥ 13 g/dL; 55.1%) was observed among those with CKD G3a, whereas the highest proportion of patients with hemoglobin values < 9 g/dL (17.2%) was observed among those with CKD G5 (Fig. 3a). Anemia prevalence (Hb ≤ 11 g/dL) was lowest among patients with CKD G3a (11.6%) and increased with higher CKD stage (G3b: 25.7%, G4: 52.7%, G5: 73.6%) (Fig. 3b).

### Anemia treatment 2022–2023

**Fig. 4:**
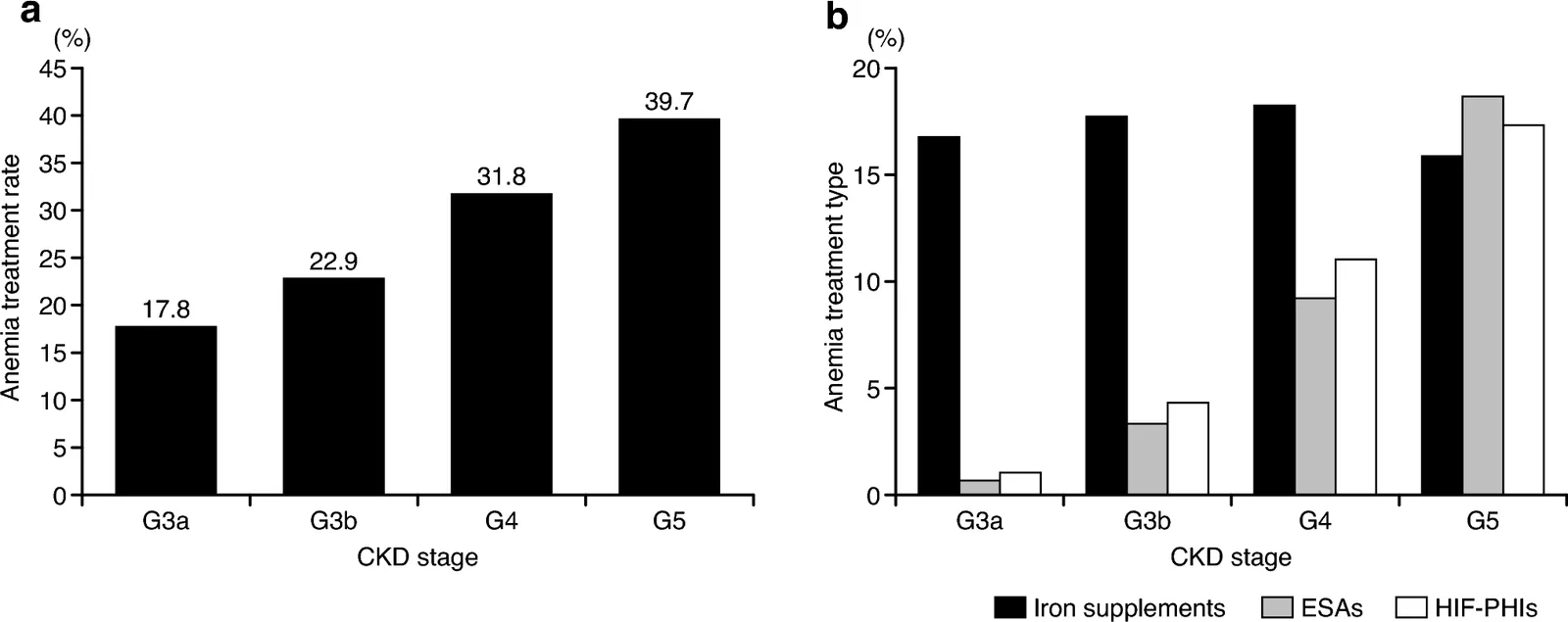
Anemia treatment rates by CKD stage between 2022 and 2023. **a** Anemia treatment rates in patients with CKD and anemia by CKD stage. **b** Anemia treatment type by CKD stage. *CKD* Chronic kidney disease, *ESAs* Erythropoiesis-Stimulating agents, *G* Grade, *HIF–PHIs* Hypoxia-Inducible factor–Prolyl hydroxylase inhibitors

Overall, anemia treatment rates increased with higher CKD stages, ranging from 17.8% (G3a) to 39.7% (G5) (Fig. 4a). Iron supplements were the most common treatment in patients with G3a, G3b, or G4 (Fig. 4b). Use of ESAs and HIF–PHIs was highest in patients with CKD G5.

Among all CKD stages, oral supplements were the most common iron supplementation (87.8%–93.9%). For ESAs, darbepoetin alfa was the most common type used (58.5%–77.7%) (Supplementary Table 5). For HIF–PHIs, daprodustat was the most common type (64.6%–69.5%).

### Anemia laboratory tests 2022–2023

Per the inclusion criteria, all patients had hemoglobin measurements, and most patients had MCV tests, regardless of CKD stage (97.8%–98.7%) (Table 2). The types of anemia-related laboratory tests were similar in patients across all CKD stages. Nearly 20% of patients had ferritin measurements (18.8%–19.2%), and few patients were tested for TSAT (1.2%–1.5%). Approximately a third of patients had iron measurements taken (31.7%–33.7%), and 11.7% to 12.9% were tested for TIBC.

**Table 2:** Percentage of anemia-related tests received by patients with CKD and anemia between 2022 and 2023

	G3a (n = 26,542)	G3b (n = 18,421)	G4 (n = 9363)	G5 (n = 2218)
Hb	100.0	100.0	100.0	100.0
MCV	97.8	98.2	98.2	98.7
Reticulocyte	4.7	4.8	5.0	5.0
Ferritin	19.2	18.8	18.8	19.2
TSAT	1.2	1.3	1.5	1.5
Iron	33.7	33.5	33.7	31.7
TIBC	12.9	11.7	12.0	12.8
Vitamin B12	2.0	1.9	2.0	1.7
Folic acid	3.3	3.6	3.4	3.1
Erythropoietin in the blood	0.9	2.0	3.1	3.0

Data shown as percentages. CKD G3a: estimated glomerular filtration rate ≥ 45 mL/min/1.73 m^2^ and < 60 mL/min/1.73 m^2^, G3b: ≥ 30 and < 45 mL/min/1.73 m^2^, G4: ≥ 15 and < 30 mL/min/1.73 m^2^, and G5: < 15 mL/min/1.73 m^2^

*CKD* Chronic kidney disease, *G* Grade, *Hb* Hemoglobin, *MCV* Mean corpuscular volume, *TSAT* Transferrin saturation, *TIBC* Total iron binding capacity

### Subgroup analyses 2022–2023

**Fig. 5:**
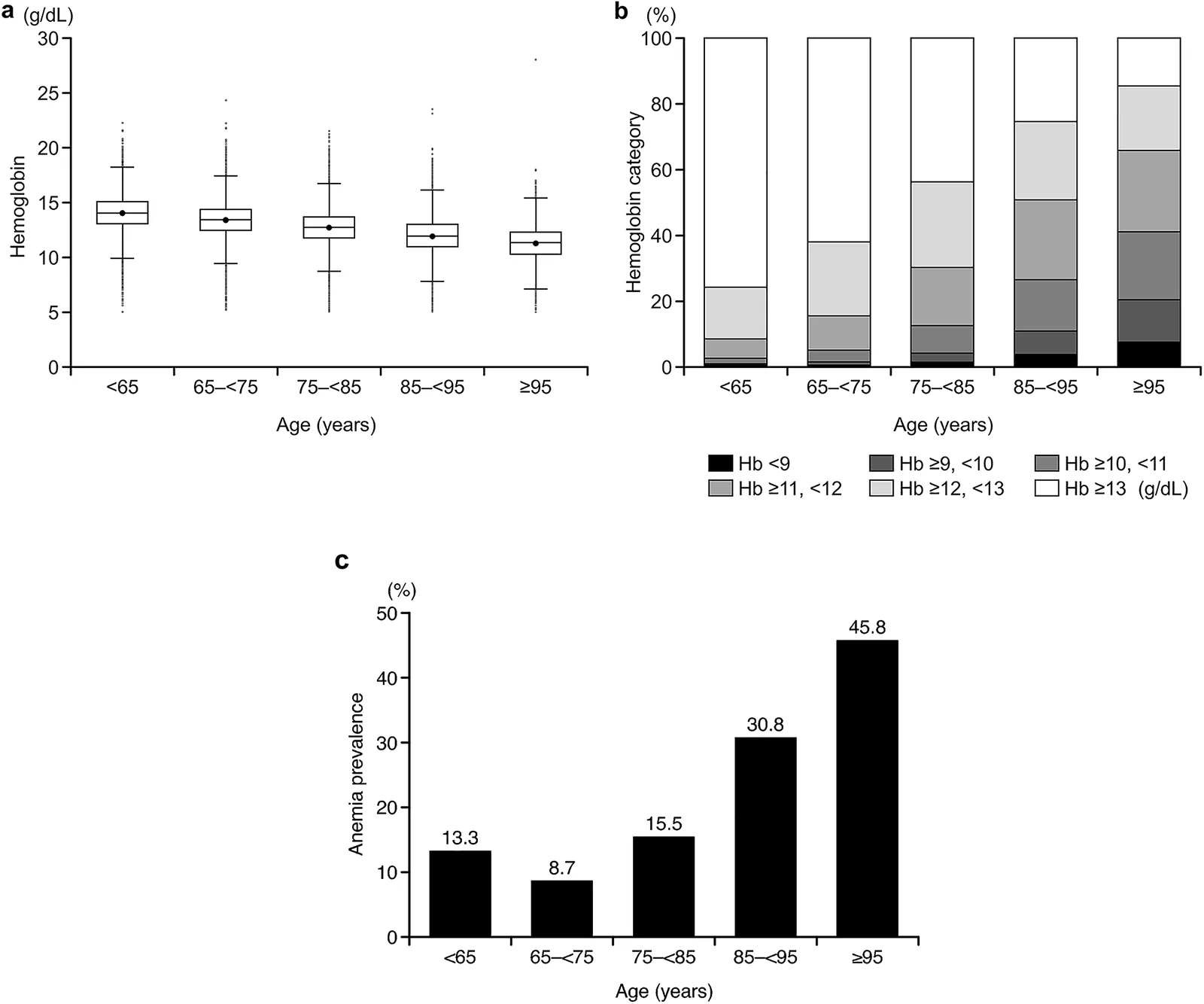
Hemoglobin levels and anemia prevalence by age between 2022 and 2023. **a** Box-and-whisker plots depict the median, interquartile range, and the minimum and maximum observations (min–max). Whiskers extend to the lowest and highest non-outlier values, while outliers are shown as individual points. **b** Distribution of hemoglobin levels by age group. **c** Anemia prevalence in patients with chronic kidney disease by age group. *Hb* Hemoglobin

**Fig. 6:**
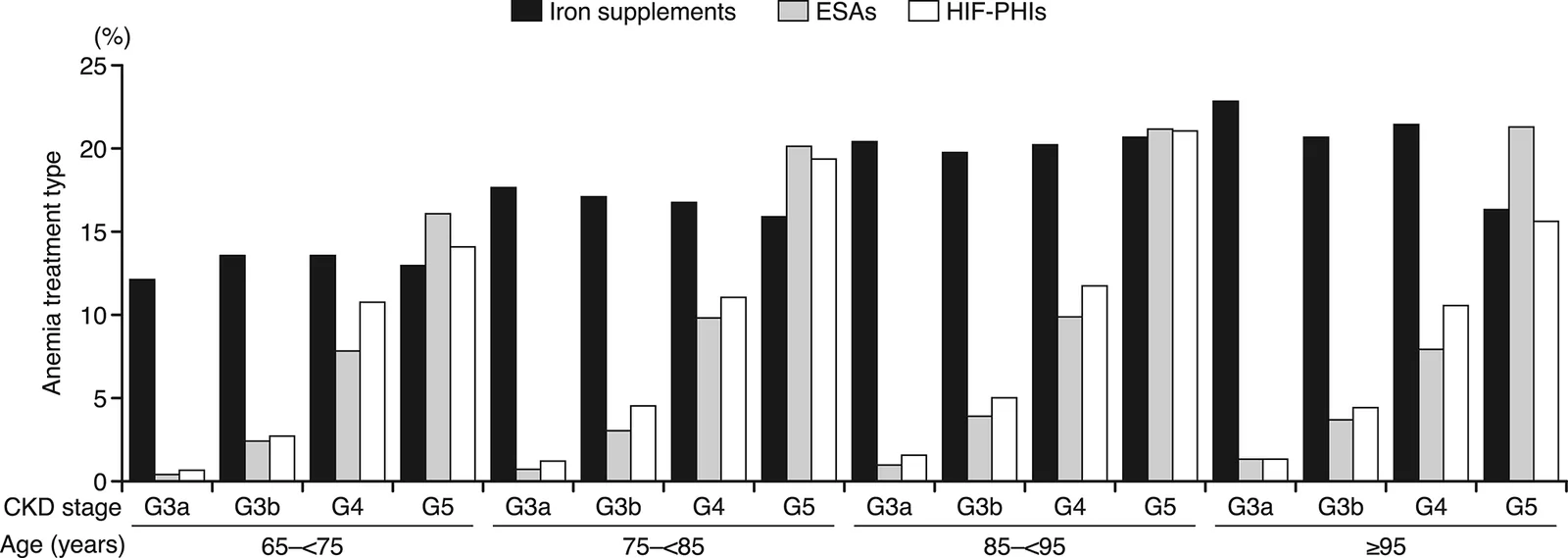
Anemia treatment type and rates by CKD stage and age between 2022 and 2023. *CKD* chronic kidney disease, *ESAs* Erythropoiesis-stimulating agents, *HIF–PHIs* Hypoxia-inducible factor–Prolyl hydroxylase inhibitors

Hemoglobin values decreased as age increased, with mean values of 14.02 g/dL in patients aged < 65 years and 11.26 g/dL in those aged ≥ 95 years (Fig. 5a). The proportion of patients with Hb < 9 g/dL increased with increasing age category (Fig. 5b). Anemia prevalence was lowest in patients aged 65–74 years (8.7%), and highest in those aged ≥ 95 years (45.8%) (Fig. 5c). Across all CKD stages, patients aged ≥ 95 years had the highest anemia prevalence rates (G3a: 34.1%, G3b: 46.3%, G4: 66.7%, and G5: 83.4%) (Table 3). Among patients aged ≥ 95 years, the percentage receiving anemia treatment by CKD stage was 24.5% (G3a), 25.7% (G3b), 32.4% (G4), and 41.8% (G5). The distribution of hemoglobin levels by CKD stage and age are shown in Supplementary Fig. 2. While the use of iron supplements increased from 65 to 95 years of age, ESA and HIF–PHI use increased until 85 years of age and remained stable thereafter (Fig. 6). Regarding the type of anemia treatment, iron supplements were the most frequently used across patients aged 65–74 years, 75–84 years, and ≥ 95 years. Across all ages, ESAs were the most used treatment in those with CKD G5.

**Table 3:** Anemia prevalence and hemoglobin levels by CKD stage and by age between 2022 and 2023

	Age category			
	65–<75 (n = 83,446)	75–<85 (n = 120,094)	85–<95 (n = 74,022)	≥ 95 (n = 8042)
Anemia prevalence (%)				
G3a	6.2	10.7	21.2	34.1
G3b	14.3	20.9	34.8	46.3
G4	35.8	47.5	59.6	66.7
G5	62.8	68.9	81.4	83.4
Hb (g/dL), mean ± SD				
G3a	13.5 ± 1.5	12.91 ± 1.5	12.2 ± 1.5	11.6 ± 1.6
G3b	13.2 ± 1.7	12.4 ± 1.6	11.7 ± 1.6	11.3 ± 1.6
G4	12.2 ± 1.9	11.5 ± 1.7	11.0 ± 1.7	10.6 ± 1.6
G5	11.2 ± 1.8	10.7 ± 1.8	10.2 ± 1.9	9.9 ± 2.2
Undergoing treatment for anemia (%)				
G3a	12.9	18.8	21.9	24.5
G3b	17.3	22.4	25.7	25.7
G4	26.3	31.2	34.5	32.4
G5	33.5	42.6	47.0	41.8

CKD G3a: estimated glomerular filtration rate ≥ 45 mL/min/1.73 m^2^ and < 60 mL/min/1.73 m^2^, G3b: ≥ 30 and < 45 mL/min/1.73 m^2^, G4: ≥ 15 and < 30 mL/min/1.73 m^2^, and G5: < 15 mL/min/1.73 m^2^. *CKD* Chronic kidney disease, *G* Grade, *Hb* Hemoglobin, *SD* Standard deviation

By sex, female patients had lower mean hemoglobin values than male patients (12.4 g/dL and 13.7 g/dL, respectively) (Supplementary Fig. 3a and 3b), higher anemia prevalence (Supplementary Fig. 3c), higher anemia treatment rate (Supplementary Fig. 3d), and higher use of iron supplements (Supplementary Fig. 3e).

**Fig. 7:**
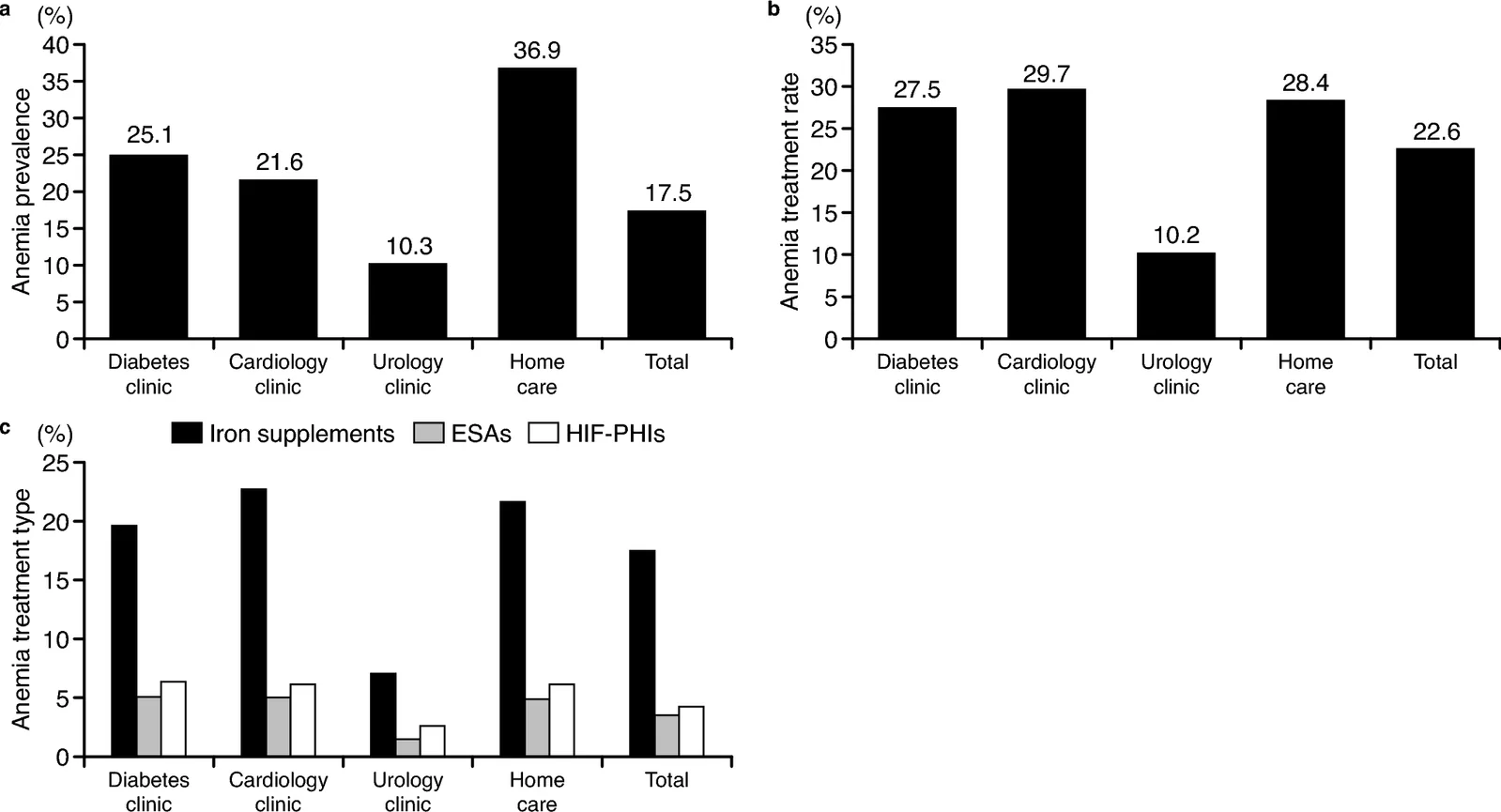
Anemia prevalence, treatment rate, and treatment type by treatment setting between 2022 and 2023. **a** Anemia prevalence in patients with chronic kidney disease by treatment setting. **b** Anemia treatment rates by treatment setting. c. Anemia treatment type by treatment setting. *ESAs* Erythropoiesis-stimulating agents, *HIF–PHIs* Hypoxia-inducible factor–Prolyl hydroxylase inhibitors

The anemia prevalence, treatment rate, and types of anemia medications by treatment setting are shown in Fig. 7, and patient background by treatment setting is shown in Supplementary Table 6. Most patients (81.0%) had a comorbid condition, and the most common comorbidities were hypertension (64.0%), dyslipidemia (52.4%), diabetes (32.1%), and heart failure (21.6%). Patients treated in home care settings and in urology clinics had the highest (36.9%) and lowest (10.3%) prevalence of anemia, respectively (Fig. 7a). Higher anemia treatment rates were found in patients treated in diabetes clinics (27.5%), cardiology clinics (29.7%), and home care settings (28.4%) (Fig. 7b). The percentage of prescriptions for ESAs and HIF–PHIs were similar to the overall population for patients treated in diabetes clinics, cardiology clinics, and home care settings, while iron supplements were the most common anemia treatment across all treatment settings (Fig. 7c).

By region (Supplementary Fig. 4), anemia prevalence was highest in the Hokkaido–Tōhoku and Chūgoku–Shikoku regions, while anemia treatment rates were highest in the Kinki and Kyushu–Okinawa regions. In Tokyo, Chūbu, Kinki, and Chūgoku–Shikoku, approximately 20% of patients had ferritin measurements (20.1%–25.9%), while a lower percentage of patients in other regions had ferritin measurements (10.4%–17.3%) (Supplementary Table 7). TSAT measurements were highest in Kinki (3.5%), and < 2% in all other regions. The percentage of iron measurements ranged from 28.0% (Hokkaido–Tōhoku) to 44.1% (Kyushu–Okinawa), and TIBC measurements ranged from 8.7% (Hokkaido–Tōhoku) to 17.2% (Chūgoku–Shikoku).

## Discussion

This study described the current anemia treatment in patients with CKD by GPs in primary care clinics, based on data from the JAMDAS database. After HIF–PHIs were introduced for non-dialysis patients in 2020, treatment rates for anemia tended to increase, with use of HIF–PHIs outpacing ESAs between 2022 and 2023. During that period, the prevalence of anemia in CKD decreased. This study revealed that HIF–PHI use by GPs in Japan has increased, leading to changes in treatment patterns and increased treatment rates, possibly contributing to the observed improvements in hemoglobin levels.

The earlier nationwide database study showed that, in 2014, patients treated in university hospitals, especially older patients, had low hemoglobin levels [6]. Our findings suggest that the introduction of HIF–PHIs positively affected the treatment of anemia in CKD between 2022 and 2023, as shown by increased treatment rates, increased use of HIF–PHIs, and higher proportion of patients with increased hemoglobin levels. Prescription of HIF–PHIs by GPs was expected to improve anemia treatment rates in CKD, and this appears to have been achieved.

A previous study reported that patients treated by non-nephrologists had lower ESA prescription rates than those treated by nephrologists [8]. In the current study, we observed an overall increase in anemia treatment rates, especially iron supplements and HIF–PHIs, possibly reflecting growing awareness among GPs regarding the importance of iron supplements if iron deficiency occurs during HIF–PHI use [14].

The JSN2018 and JSDT2015 guidelines recommend targeting hemoglobin values of 11–13 g/dL for non-dialysis patients with CKD [13, 15]. We found that the proportion of patients who achieved these targets decreased as CKD progressed, which is consistent with previous reports. The hemoglobin values of patients in our study were low, based on the standard treatment target range of 11–13 g/dL.

Guidelines also recommend measuring ferritin and TSAT when prescribing anemia treatments [13–15], yet we observed that only ~ 20% of patients were tested for ferritin and a low proportion of patients were tested for TSAT (1.2%–1.5%), regardless of CKD stage. TSAT can be calculated from the TIBC and iron measurements. In our study, iron was measured in 31.7%–33.7% of patients, while TIBC was measured in 11.7%–12.9% of patients (approximately half the proportion measured for ferritin). However, it was unknown whether the GP calculated and monitored the TSAT values separately. In a recent survey of GPs in Osaka Prefecture, TSAT was tested half as much as ferritin before and after prescription of HIF–PHIs, and neither test was sufficiently performed [16]. Thus, the low proportion of TIBC measurements we observed may reflect the low proportion of TSAT tests. This is a critical issue, as patients managed by GPs are often elderly and have multiple comorbidities, necessitating appropriate iron-related testing to ensure accurate evaluation and anemia management.

Hemoglobin levels tended to decrease with increasing age, with a higher proportion of patients with hemoglobin < 9 g/dL increasing among older patients. Lower hemoglobin levels may have been intentionally targeted in older patients because of their physical activity levels and/or treatment priorities. Alternatively, the apparent lack of optimal target hemoglobin levels for older patients may have led to variable treatment, suggesting that there may be a need to build awareness of the adverse effects of significantly low hemoglobin.

In contrast to a report from a university hospital setting [6] that suggested that patients aged ≥ 85 years were less likely to receive anemia treatment, our findings did not show any evidence that anemia treatment was withheld in patients aged 85–94 years in primary care settings. Thus, GPs may be more inclined to provide anemia treatment to older patients. There are several potential explanations for this. For example, older patients in primary care settings may be more aware of declining quality of life and activities of daily living, and the availability of HIF–PHIs and treatment guidelines may facilitate decision-making by GPs. We observed that HIF–PHIs were used more commonly than ESAs, except in patients with CKD G5 and particularly those aged ≥ 95 years; however, interpretation is limited by the small sample size. This likely reflects treatment choices based on drug characteristics, drug adherence, and polypharmacy concerns in older patients.

A recent survey found that cardiologists and diabetes specialists in Japan use HIF–PHIs to treat anemia in CKD [17], aligning with our findings that patients received anemia treatment in both cardiology and diabetes clinics. As many patients with CKD also have diabetes and cardiovascular disease [2, 18], GPs are more likely to encounter anemia in CKD in the context of managing these primary conditions. In home care settings, both the anemia prevalence (36.9%) and treatment rates (28.4%) were relatively high. This likely reflects that patients with advanced CKD (typically managed in tertiary care institutions) are treated by GPs because of their age and multimorbidity. As Japan’s population ages, such cases are expected to become increasingly common. To support the appropriate use of HIF–PHIs and raise awareness of the relevant guidelines, wider collaboration and communication between specialists and GPs who manage CKD in the context of comorbidities or home care may be needed.

When evaluating the results by region, we observed some variations, suggesting the presence of geographical differences. While this study presents data integrated at the national level, it is important to interpret the findings considering regional variations in GP practice.

This study had several limitations. First, while the JAMDAS database contains data from a given institution, it does not capture tests or treatment performed elsewhere; thus, the connection between testing and treatment could not be analyzed, and testing or treatment rates may have been underestimated. An additional analysis showed that the proportion of untreated patients without anemia decreased with worsening CKD stage (Supplementary Table 8), suggesting that missing treatment data may have had a limited impact on the results. Second, treatment setting classifications were based on insurance claims data generated at the facility level for medical procedures; therefore, subgroup analyses by treatment setting may not accurately reflect the specialties of each doctor. Third, patients with anemia from bleeding events such as gastrointestinal bleeding or other non-CKD causes (e.g., liver cirrhosis or bone marrow disorders) were not excluded. However, these cases are less common in primary care settings and likely have minimal impact on the findings. Fourth, as the index date was defined as the last available hemoglobin measurement, it may not correspond to the timing of anemia diagnosis or treatment initiation. However, we partially mitigated this limitation by determining treatment status before the index date. Fifth, erythropoietin < 50 mIU/mL was included as one of the criteria for anemia. Although this is not a standalone indicator according to current guidelines, erythropoietin testing in patients with CKD generally reflects suspected renal anemia in routine clinical practice. In the present study, the number of patients classified as anemic solely based on erythropoietin < 50 mIU/mL was very small (110 of 397,419 patients between 2018 and 2023), and exclusion of these patients had minimal impact on the overall prevalence estimates. Nevertheless, some degree of misclassification may remain. Sixth, the data in the JAMDAS database reflect approximately 5% of primary care practices and may not be representative of all practices in Japan. Finally, as JAMDAS is a Japanese database, the study findings may not apply to other countries. Nevertheless, the key strength of the study is the large sample size, which enabled subgroup analyses, including a description of the actual treatment practices in older patients. Our study provides important insights regarding changes in treatment practices since the launch of HIF–PHIs in Japan.

In conclusion, this study revealed current treatment patterns for anemia in patients with CKD in Japan and found that the use of HIF–PHIs by GPs is increasing, reflecting a shift in treatment practices in primary care. Further efforts are necessary to ensure that treatment practices align with guideline recommendations, particularly for those with CKD stages G4 or G5. Future research should focus on anemia in older patients with CKD, as most patients with CKD in primary care clinics in Japan are older and have low hemoglobin levels, with limited evidence to guide treatment.

## Supplementary Information

Below is the link to the electronic supplementary material.

Supplementary file1 (DOCX 250 kb)
