# Supplementary material for: Anemia prevalence and treatment patterns in chronic kidney disease: a nationwide observational study using a Japanese primary care database (2018–2023)
Source: Clin Exp Nephrol. 2026 Jun 30;30(9):1292–302. doi: 10.1007/s10157-026-02873-2 (PMC13518424; doi:10.1007/s10157-026-02873-2)
Supplement: Supplementary file 1 [file 10157_2026_2873_MOESM1_ESM.docx]

**Supplementary material**

**Supplementary Fig. 1** Dataset flow diagram between 2018 and 2023

CKD, chronic kidney disease; eGFR, estimated glomerular filtration rate; G, grade; NDD, non-dialysis-dependent.

**Supplementary Fig. 2** Hemoglobin level distribution by CKD stage and age between 2022 and 2023

CKD G3a: estimated glomerular filtration rate ≥45 mL/min/1.73 m2 and <60 mL/min/1.73 m2, G3b: ≥30 and <45 mL/min/1.73 m2, G4: ≥15 and <30 mL/min/1.73 m2, and G5: <15 mL/min/1.73 m2.

CKD, chronic kidney disease; G, grade; Hb, hemoglobin.

**Supplementary Fig. 3** Hemoglobin levels and anemia treatment type by sex between 2022 and 2023

a. Box-and-whisker plots depict the median, interquartile range, and the minimum and maximum observations (min–max). Whiskers extend to the lowest and highest non-outlier values, while outliers are shown as individual points. b. Distribution of hemoglobin levels by sex. c. Anemia prevalence by sex. d. Treatment rate by sex. e. Anemia treatment type by sex.

ESAs, erythropoiesis-stimulating agents; Hb, hemoglobin; HIF–PHIs, hypoxia-inducible factor–prolyl hydroxylase inhibitors.

**Supplementary Fig. 4** Anemia prevalence, treatment rate, and treatment type by region between 2022 and 2023

a. Anemia prevalence in patients with chronic kidney disease by region. b. Anemia treatment rates by region. c. Anemia treatment type by region. Hokkaido-Tōhoku includes Hokkaido, Aomori, Akita, Iwate, Yamagata, Miyagi, and Fukushima. Kanto includes Tochigi, Ibaraki, Gunma, Chiba, Saitama, and Kanagawa. Tokyo includes Tokyo. Chūbu includes Niigata, Nagano, Yamanashi, Toyama, Ishikawa, Fukui, Gifu, Shizuoka, and Aichi. Kinki includes Shiga, Mie, Kyoto, Hyogo, Osaka, Nara, and Wakayama. Chūgoku–Shikoku includes Okayama, Shimane, Tottori, Hiroshima, Yamaguchi, Kagawa, Tokushima, Ehime, and Kochi. Kyushu–Okinawa includes Fukuoka, Oita, Saga, Nagasaki, Miyazaki, Kumamoto, Kagoshima, and Okinawa.

ESAs, erythropoiesis-stimulating agents; HIF–PHIs, hypoxia-inducible factor–prolyl hydroxylase inhibitors.

**Supplementary Table 1** Codes used to identify diagnoses, medications, and tests

| **Category** | **Description** | **Code** |
| --- | --- | --- |
| **Exclusion criteria** |  |  |
| Diagnosis of acute renal failure | Acute kidney injury | ICD-10 code: N00.x |
|  | Rapidly progressive glomerulonephritis | ICD-10 code: N01.x |
|  | Acute kidney failure | ICD-10 code: N17.x |
|  | Pregnancy-related acute kidney injury | ICD-10 codes: O03.32, O03.82, O04.82, O07.32, O08.4, or O90.4 |
|  | Traumatic anuria | ICD-10 code: T79.5 |
| Pregnancy record | Pregnancy-related additional medical fee (abolished in 2020) | Electronic receipt code: 820100348 |
|  | Pregnancy ending in miscarriage | ICD-10 codes: O00–O05 |
|  | Edema, proteinuria, and hypertensive disorders during pregnancy, childbirth, and the postpartum period | ICD-10 codes: O10–O16 |
|  | Other obstetric conditions | ICD-10 codes: O94–O98, O99.0, or O99.1 |
| Diagnosis of end-stage renal failure | Hemodialysis, peritoneal dialysis | Electronic receipt codes: 150196310, 150338610, 190167970, 113002510, 114009310, 114009410, 114009510, 140057810, 140057910, 140058010, 140059310, 140059410, 140059510, 140058110, 140058210, 140058310, 140058410, 140058510, 140058610, 140036710, 140051010, 140051110, or 140052810 |
|  | Post-kidney transplant condition | ICD-10 codes: Z94.0 or T86.1 |
| Diagnosis of malignant tumor | Malignant neoplasms | ICD-10 codes: C00–C97 |
| **Patient background** |  |  |
| Comorbidities | Hypertension | ICD-10 codes: I10–I15 |
|  | Ischemic heart disease | ICD-10 codes: I20–I25 |
|  | Peripheral artery disease | ICD-10 codes: I70.2, I73.9, I74.3, or I74.4 |
|  | Stroke | ICD-10 codes: I60–I69 |
|  | Heart failure | ICD-10 codes: I11.0, I13.0, I13.2, or I50 |
|  | Diabetes | ICD-10 codes: E11–E14 |
|  | Dyslipidemia | ICD-10 codes: E78.0–E78.5 |
| Blood tests | Hemoglobin | JAMDAS code: 115_1 |
|  | eGFR | Calculated from serum creatinine (JAMDAS code: 160_1) |
|  | Serum albumin | JAMDAS code: 194_1 |
|  | Uric acid | JAMDAS code: 70_1 |
|  | Sodium | JAMDAS code: 136_1 |
|  | Potassium | JAMDAS code: 169_1 |
|  | Chloride | JAMDAS code: 157_1 |
|  | Phosphate | JAMDAS code: 49_1 |
|  | Calcium | JAMDAS code: 168_1 |
|  | C-reactive protein | JAMDAS code: 234_1 |
| Urine tests | Proteinuria (quantitative) | JAMDAS code: 72_3 |
|  | Proteinuria (qualitative) | JAMDAS code: 72_4 |
|  | Urinary albumin | JAMDAS code: 194_3 |
| **Prevalence of anemia** |  |  |
| Anemia diagnosis | Nutritional anemia (including iron deficiency anemia, vitamin B12 deficiency anemia) | ICD-10 codes: D50–D53 |
|  | Hemolytic anemia (including enzyme deficiency anemia, sickle cell anemia) | ICD-10 codes: D55–D59 |
|  | Aplastic anemia and other anemias | ICD-10 codes: D60–D61 |
|  | Renal anemia | ICD-10 codes: D63.8 or N19; electronic receipt code: 2858001 |
| **Type of anemia medication** |  |  |
| Iron supplements | Intravenous iron |  |
|  | Saccharated ferric oxide | Electronic receipt code: 620005208 |
|  | Ferric carboxymaltose | Electronic receipt code: 622676601 |
|  | Ferric derisomaltose | Electronic receipt codes: 622926501 or 622926601 |
|  | Oral iron supplements |  |
|  | Dried ferrous sulfate | ATC code: B03AA07 |
|  | Sodium ferrous citrate | ATC code: B03AA12 |
|  | Ferrous fumarate | ATC code: B03AA02 |
|  | Ferrous pyrophosphate | ATC code: 620005924 |
|  | Iron-based phosphate binder |  |
|  | Ferric citrate hydrate | ATC code: V03AE08 |
| ESAs | Epoetin | ATC code: B03XA01 |
|  | Darbepoetin alfa | ATC code: B03XA02 |
|  | Epoetin beta pegol | ATC code: B03XA03 |
| HIF–PHIs | Roxadustat | ATC code: B03XA05 |
|  | Daprodustat | ATC code: B03XA07 |
|  | Vadadustat | ATC code: B03XA08 |
|  | Enarodustat | Electronic receipt codes: 622829501 or 622829601 |
|  | Molidustat sodium | Electronic receipt codes: 622854901, 622855001, 622855101, or 622855301 |
| **Anemia-related laboratory tests** |  |  |
| Tests | Hemoglobin | JAMDAS code: 115_1 |
|  | Mean corpuscular volume | JAMDAS code: 218_1 |
|  | Reticulocyte | JAMDAS codes: 17_1 or 17_2 |
|  | Ferritin | JAMDAS code: 123_1 |
|  | Transferrin saturation | JAMDAS code: 278_1 |
|  | Iron | JAMDAS code: 10_1 |
|  | Vitamin B12 | JAMDAS code: 282_1 |
|  | Folic acid | JAMDAS code: 281_1 |
|  | Erythropoietin | JAMDAS code: 280_1 |
|  | Total iron-binding capacity | JAMDAS code: 35_1 |

ATC, Anatomical Therapeutic Chemical; eGFR, estimated glomerular filtration rate; ESAs, erythropoiesis-stimulating agents; HIF–PHIs, hypoxia-inducible factor–prolyl hydroxylase inhibitors; ICD-10, International Classification of Diseases 10th Revision; JAMDAS, Japan Medical Data Survey.

**Supplementary Table 2** Definition of the treatment setting

| **Category** | **Definition** |
| --- | --- |
| Diabetes clinic | Medical institutions specializing in diabetes internal medicine, or medical institutions that meet any of the following criteria:   - Blood glucose self-monitoring device surcharge - Diabetes complication management fee - Diabetes dialysis prevention guidance and management fee   Medical institutions that charge a fee for artificial kidneys (chronic maintenance dialysis) were excluded from this category. |
| Cardiology clinic | Medical institutions specializing in cardiology or medical institutions that perform Holter monitoring or echocardiography |
| Urology clinic | Medical institutions specializing in urology |
| Home care | Patients who meet any of the following criteria:   - Home visit medical care fee (home visit fee) - Comprehensive medical management fee for home care or comprehensive medical management fee for facility admission - Home care guidance and management fee |

**Supplementary Table 3** Anemia medication prescriptions by type between 2018 and 2023

|  | **2018** | **2019** | **2020** | **2021** | **2022** | **2023** |
| --- | --- | --- | --- | --- | --- | --- |
| **Iron supplements** |  |  |  |  |  |  |
| Patients (n) | 129 | 259 | 493 | 961 | 1671 | 8757 |
| Intravenous iron (%) | 7.8 | 6.9 | 7.5 | 8.8 | 8.9 | 6.9 |
| Oral iron (%) | 91.5 | 95.4 | 94.3 | 93.8 | 92.8 | 93.0 |
| Iron-containing phosphate binder (%) | 1.6 | 0.0 | 0.4 | 0.1 | 1.0 | 2.3 |
| **ESAs** |  |  |  |  |  |  |
| Patients (n) | 48 | 123 | 217 | 378 | 441 | 1645 |
| Epoetin (%) | 6.3 | 8.1 | 2.3 | 2.4 | 1.8 | 1.2 |
| Darbepoetin alfa (%) | 35.4 | 43.1 | 53.5 | 65.3 | 65.3 | 61.4 |
| Epoetin beta pegol (%) | 58.3 | 52.0 | 49.3 | 33.3 | 34.2 | 39.1 |
| **HIF–PHIs** |  |  |  |  |  |  |
| Patients (n) | 0 | 0 | 1 | 80 | 356 | 2161 |
| Roxadustat (%) | - | - | 0.0 | 23.8 | 12.9 | 9.2 |
| Daprodustat (%) | - | - | 100.0 | 53.8 | 62.9 | 66.0 |
| Vadadustat (%) | - | - | 0.0 | 18.8 | 21.3 | 19.9 |
| Enarodustat (%) | - | - | 0.0 | 0.0 | 0.6 | 2.2 |
| Molidustat (%) | - | - | 0.0 | 3.8 | 3.7 | 4.5 |

Patients may have received more than one type of treatment (including ESAs + iron supplements and HIF–PHIs + iron supplements).
ESAs, erythropoiesis-stimulating agents; HIF–PHIs, hypoxia-inducible factor–prolyl hydroxylase inhibitors.

**Supplementary Table 4** Patient characteristics by CKD stages between 2022 and 2023

|  |  | **G3a**  **(n = 228,836)** | **G3b (n = 71,742)** | **G4**  **(n = 17,783)** | **G5**  **(n = 3015)** |
| --- | --- | --- | --- | --- | --- |
| Sex (%) | Male | 40.9 | 39.7 | 38.8 | 51.5 |
| Age (years) | Mean ± SD | 74.58 ± 11.48 | 80.89 ± 9.79 | 83.09 ± 10.07 | 79.13 ± 12.48 |
| Age category (%) | <65 | 18.9 | 6.6 | 5.4 | 12.9 |
|  | 65–74 | 27.7 | 17.1 | 12.3 | 18.7 |
|  | 75–84 | 35.1 | 38.3 | 32.4 | 30.6 |
|  | 85–94 | 16.9 | 33.8 | 42.2 | 32.1 |
|  | ≥95 | 1.4 | 4.2 | 7.7 | 5.6 |
| BMI (kg/m^2^) | Mean ± SD | 23.51 ± 3.68 | 23.46 ± 3.75 | 23.42 ± 3.95 | 22.99 ± 3.89 |
| Comorbidity (%) | Hypertension | 60.7 | 72.0 | 76.3 | 71.7 |
|  | Diabetes | 29.3 | 35.7 | 40.1 | 46.0 |
|  | Dyslipidemia | 52.3 | 52.2 | 49.7 | 42.2 |
|  | Heart failure | 16.8 | 31.0 | 45.9 | 40.5 |
|  | Ischemic heart disease | 12.6 | 18.9 | 23.2 | 20.7 |
|  | Stroke | 12.0 | 16.0 | 18.2 | 16.8 |
|  | Peripheral arterial disease | 1.4 | 1.8 | 1.9 | 1.9 |
| **Blood assessments** |  |  |  |  |  |
| eGFR (mL/min/1.73 m^2^) | Mean ± SD | 53.65 ± 4.17 | 39.12 ± 4.16 | 24.38 ± 4.07 | 10.59 ± 2.86 |
| Serum albumin (g/dL) | Mean ± SD | 4.08 ± 0.40 | 3.91 ± 0.46 | 3.71 ± 0.52 | 3.52 ± 0.63 |
| Uric acid (mg/dL) | Mean ± SD | 5.53 ± 1.29 | 6.09 ± 1.44 | 6.65 ± 1.84 | 6.46 ± 2.30 |
| Sodium (mEq/L) | Mean ± SD | 141.2 ± 2.6 | 140.8 ± 3.1 | 140.2 ± 3.7 | 139.5 ± 4.5 |
| Potassium (mEq/L) | Mean ± SD | 4.23 ± 0.50 | 4.36 ± 0.57 | 4.56 ± 0.70 | 4.76 ± 0.89 |
| Chloride (mEq/L) | Mean ± SD | 103.8 ± 3.0 | 104.0 ± 3.6 | 104.3 ± 4.9 | 104.6 ± 6.3 |
| Phosphate (mg/dL) | Mean ± SD | 3.32 ± 0.57 | 3.38 ± 0.60 | 3.54 ± 0.66 | 4.27 ± 1.07 |
| Calcium (mg/dL) | Mean ± SD | 9.14 ± 0.44 | 9.12 ± 0.53 | 9.01 ± 0.70 | 8.61 ± 0.91 |
| C-reactive protein (mg/dL) | Median (IQR) | 0.10 (0.05, 0.28) | 0.13 (0.06, 0.51) | 0.21 (0.07, 1.07) | 0.35 (0.09, 2.23) |
| **Urine assessments** |  |  |  |  |  |
| Urine protein (quantitative) (mg/dL) | Median (IQR) | 8.0 (5.0, 19.0) | 12.0 (5.0, 36.0) | 32.5 (11.3, 83.0) | 69.0 (38.0, 191.0) |
| Urine protein (qualitative) (%) | Negative | 5.7 | 4.4 | 2.3 | 0.4 |
|  | ± | 1.0 | 1.1 | 0.8 | 0.6 |
|  | 1+ | 0.6 | 1.0 | 1.3 | 1.6 |
|  | ≥2+ | 0.3 | 0.8 | 2.0 | 4.1 |
| Urinary albumin (mg/dL) | Median (IQR) | 15.5 (6.7, 45.2) | 29.6 (10.4, 124.3) | 73.9 (22.3, 383.0) | 891.0 (94.1, 2302.0) |

CKD G3a: eGFR ≥45 mL/min/1.73 m^2^ and <60 mL/min/1.73 m^2^, G3b: ≥30 and <45 mL/min/1.73 m^2^, G4: ≥15 and <30 mL/min/1.73 m^2^, and G5: <15 mL/min/1.73 m^2^.

BMI, body mass index; CKD, chronic kidney disease; eGFR, estimated glomerular filtration rate; G, grade; IQR, interquartile range; SD, standard deviation.

**Supplementary Table 5** Anemia medication prescriptions by type, by CKD stage, between 2022 and 2023

|  | **G3a** | **G3b** | **G4** | **G5** | **Total** |
| --- | --- | --- | --- | --- | --- |
| **Iron supplements** |  |  |  |  |  |
| Patients (n) | 4449 | 3263 | 1707 | 352 | 10,428 |
| Intravenous iron (%) | 7.4 | 6.6 | 6.6 | 7.7 | 7.2 |
| Oral iron (%) | 93.1 | 93.9 | 93.1 | 87.8 | 93.0 |
| Iron-containing phosphate binder (%) | 1.8 | 1.5 | 2.5 | 6.5 | 2.1 |
| **ESAs** |  |  |  |  |  |
| Patients (n) | 175 | 612 | 862 | 414 | 2086 |
| Epoetin (%) | 1.1 | 1.3 | 1.5 | 1.2 | 1.3 |
| Darbepoetin alfa (%) | 77.7 | 62.6 | 61.0 | 58.5 | 62.2 |
| Epoetin beta pegol (%) | 21.7 | 37.7 | 38.7 | 43.2 | 38.1 |
| **HIF–PHIs** |  |  |  |  |  |
| Patients (n) | 275 | 797 | 1033 | 384 | 2517 |
| Roxadustat (%) | 8.4 | 9.7 | 10.6 | 8.6 | 9.7 |
| Daprodustat (%) | 69.5 | 65.1 | 64.6 | 66.7 | 65.6 |
| Vadadustat (%) | 15.3 | 20.6 | 20.6 | 21.6 | 20.1 |
| Enarodustat (%) | 2.5 | 1.9 | 1.9 | 1.8 | 2.0 |
| Molidustat (%) | 6.5 | 4.1 | 3.7 | 4.2 | 4.4 |

Patients may have received more than one type of treatment (including ESAs + iron supplements and HIF–PHIs + iron supplements). CKD G3a: estimated glomerular filtration rate ≥45 mL/min/1.73 m^2^ and <60 mL/min/1.73 m^2^, G3b: ≥30 and <45 mL/min/1.73 m^2^, G4: ≥15 and <30 mL/min/1.73 m^2^, and G5: <15 mL/min/1.73 m^2^.
CKD, chronic kidney disease; ESAs, erythropoiesis-stimulating agents; G, grade; HIF–PHIs, hypoxia-inducible factor–prolyl hydroxylase inhibitors.

**Supplementary Table 6** Patient background by treatment setting between 2022 and 2023

|  |  | **All patients**  **(N = 340,398)** | **Diabetes clinic**  **(n = 6916)** | **Cardiology clinic (n = 26,962)** | **Urology clinic**  **(n = 6663)** | **Home care**  **(n = 33,108)** |
| --- | --- | --- | --- | --- | --- | --- |
| Sex | Male | 139,136 (40.9) | 3732 (54.0) | 11,509 (42.7) | 4935 (74.1) | 12,911 (39.0) |
| Age (years) | Mean ± SD | 76.24 ± 11.63 | 73.96 ± 11.32 | 78.17 ± 9.95 | 73.88 ± 11.57 | 81.99 ± 11.72 |
| Age category (%) | <65 | 16.1 | 19.9 | 10.1 | 20.7 | 9.6 |
|  | 65–74 | 24.5 | 28.8 | 22.8 | 27.2 | 13.9 |
|  | 75–84 | 35.3 | 35.2 | 40.5 | 35.4 | 27.3 |
|  | 85–94 | 21.7 | 15.3 | 24.8 | 15.9 | 39.8 |
|  | ≥95 | 2.4 | 0.8 | 1.8 | 0.8 | 9.4 |
|  |  |  |  |  |  |  |
| BMI | Mean ± SD (kg/m^2^) | 23.50 ± 3.72 | 24.16 ± 3.94 | 23.48 ± 3.66 | 23.52 ± 3.99 | 23.63 ± 4.38 |
| Comorbidity (%) | Hypertension | 64.0 | 77.7 | 81.7 | 24.0 | 72.8 |
|  | Ischemic heart disease | 14.6 | 26.4 | 31.4 | 2.5 | 21.9 |
|  | Peripheral arterial disease | 1.5 | 2.8 | 2.2 | 0.4 | 1.9 |
|  | Stroke | 13.3 | 19.1 | 20.8 | 2.6 | 21.7 |
|  | Heart failure | 21.6 | 25.3 | 49.8 | 4.0 | 42.0 |
|  | Diabetes | 32.1 | 90.4 | 38.9 | 12.6 | 45.6 |
|  | Dyslipidemia | 52.4 | 70.9 | 62.6 | 24.2 | 47.3 |
| **Blood assessments** |  |  |  |  |  |  |
| eGFR | Mean ± SD (mL/min/1.73 m^2^) | 49.34 ± 10.66 | 46.87 ± 13.55 | 48.27 ± 10.97 | 49.75 ± 10.22 | 45.69 ± 13.76 |
| Serum albumin | Mean ± SD (g/dL) | 4.01 ± 0.44 | 3.95 ± 0.49 | 4.02 ± 0.41 | 4.07 ± 0.38 | 3.65 ± 0.57 |
| Uric acid | Mean ± SD (mg/dL) | 5.69 ± 1.41 | 5.68 ± 1.45 | 5.75 ± 1.43 | 5.80 ± 1.31 | 5.74 ± 1.65 |
| Sodium | Mean ± SD (mEq/L) | 141.0 ± 2.9 | 140.2 ± 3.2 | 141.0 ± 2.9 | 140.9 ± 2.7 | 140.2 ± 3.9 |
| Potassium | Mean ± SD (mEq/L) | 4.28 ± 0.55 | 4.36 ± 0.57 | 4.27 ± 0.53 | 4.33 ± 0.54 | 4.25 ± 0.67 |
| Chloride | Mean ± SD (mEq/L) | 103.8 ± 3.4 | 103.0 ± 4.1 | 103.8 ± 3.5 | 103.7 ± 3.0 | 103.1 ± 4.5 |
| Phosphate | Mean ± SD (mg/dL) | 3.35 ± 0.60 | 3.41 ± 0.65 | 3.36 ± 0.56 | 3.15 ± 0.62 | 3.41 ± 0.65 |
| Calcium | Mean ± SD (mg/dL) | 9.12 ± 0.49 | 8.98 ± 0.53 | 9.10 ± 0.48 | 9.06 ± 0.43 | 8.88 ± 0.61 |
| C-reactive protein | Median (mg/dL) | 0.110 (0.050, 0.370) | 0.140 (0.060, 0.560) | 0.100 (0.050, 0.290) | 0.100 (0.050, 0.390) | 0.220 (0.071, 1.140) |
| **Urine assessments** |  |  |  |  |  |  |
| Urine protein (quantitative) (mg/dL) | Median (IQR) | 11.0 (5.0, 32.0) | 62.0 (25.5, 194.0) | 12.0 (6.0, 24.0) | 19.0 (8.0, 59.0) | 59.5 (17.3, 194.0) |
| Urine protein (qualitative) (%) | Negative | 5.4 | 14.0 | 6.2 | 15.3 | 3.8 |
|  | ± | 1.0 | 3.8 | 1.0 | 3.2 | 1.1 |
|  | 1+ | 0.7 | 3.9 | 0.8 | 2.1 | 1.1 |
|  | ≥2+ | 0.5 | 3.9 | 0.6 | 1.3 | 1.1 |
| Urinary albumin (mg/dL) | Median (IQR) | 17.7 (7.2, 57.5) | 26.0 (8.4, 92.8) | 17.0 (7.2, 44.8) | 105.2 (23.9, 303.2) | 23.6 (7.9, 107.2) |

Data shown as n (%) unless otherwise stated.

BMI, body mass index; eGFR, estimated glomerular filtration rate; IQR, interquartile range; SD, standard deviation.

**Supplementary Table 7** Percentage of anemia-related tests received by patients with CKD and anemia by region between 2022 and 2023

|  | **Hokkaido–Tōhoku^*^**  **(n = 12,065)** | **Kanto**  **(n = 74,308)** | **Tokyo**  **(n = 40,777)** | **Chūbu**  **(n = 81,182)** | **Kinki**  **(n = 67,304)** | **Chūgoku–Shikoku**  **(n = 23,356)** | **Kyushu–Okinawa**  **(n = 41,406)** |
| --- | --- | --- | --- | --- | --- | --- | --- |
| Hb | 100.0 | 100.0 | 100.0 | 100.0 | 100.0 | 100.0 | 100.0 |
| MCV | 99.6 | 97.8 | 94.9 | 97.8 | 99.1 | 98.3 | 99.6 |
| Reticulocyte | 5.0 | 3.2 | 5.8 | 6.4 | 4.0 | 8.4 | 2.6 |
| Ferritin | 10.4 | 17.3 | 25.9 | 21.2 | 20.2 | 20.1 | 13.2 |
| TSAT | 0.1 | 0.4 | 0.0 | 1.9 | 3.5 | 0.3 | 0.6 |
| Iron | 28.0 | 27.3 | 30.3 | 36.1 | 34.1 | 33.1 | 44.1 |
| TIBC | 8.7 | 10.8 | 13.2 | 15.7 | 10.5 | 17.2 | 9.6 |
| Vitamin B12 | 1.3 | 2.3 | 3.6 | 1.9 | 0.4 | 1.7 | 3.2 |
| Folic acid | 0.5 | 2.3 | 3.1 | 5.1 | 3.0 | 4.2 | 3.3 |
| Erythropoietin in the blood | 1.2 | 1.5 | 1.4 | 1.8 | 2.3 | 1.9 | 0.9 |

Data shown as %. CKD G3a: estimated glomerular filtration rate ≥45 mL/min/1.73 m^2^ and <60 mL/min/1.73 m^2^, G3b: ≥30 and <45 mL/min/1.73 m^2^, G4: ≥15 and <30 mL/min/1.73 m^2^, and G5: <15 mL/min/1.73 m^2^.

^*^Definition of region classification

Hokkaido–Tōhoku includes Hokkaido, Aomori, Akita, Iwate, Yamagata, Miyagi, and Fukushima. Kanto includes Tochigi, Ibaraki, Gunma, Chiba, Saitama, and Kanagawa. Tokyo includes Tokyo. Chūbu includes Niigata, Nagano, Yamanashi, Toyama, Ishikawa, Fukui, Gifu, Shizuoka, and Aichi. Kinki includes Shiga, Mie, Kyoto, Hyogo, Osaka, Nara, and Wakayama. Chūgoku–Shikoku includes Okayama, Shimane, Tottori, Hiroshima, Yamaguchi, Kagawa, Tokushima, Ehime, and Kochi. Kyushu–Okinawa includes Fukuoka, Oita, Saga, Nagasaki, Miyazaki, Kumamoto, Kagoshima, and Okinawa.

CKD, chronic kidney disease; G, grade; Hb, hemoglobin; MCV, mean corpuscular volume; TSAT, transferrin saturation; TIBC, total iron binding capacity.

**Supplementary Table 8** Percentage of untreated patients with and without anemia by CKD stage

|  | **G3a** | **G3b** | **G4** | **G5** |
| --- | --- | --- | --- | --- |
| Patients not receiving anemia treatment | 224,105 | 67,518 | 14,803 | 2,135 |
| Without anemia | 209,776 (93.6) | 56,554 (83.8) | 9,321 (63.0) | 883 (41.4) |
| With anemia | 14,329 (6.4) | 10,964 (16.2) | 5,482 (37.0) | 1,252 (58.6) |

Data shown as n or n (%). Anemia was classified according to the JSDT anemia criteria, which uses age-specific hemoglobin thresholds.

CKD, chronic kidney disease; G, grade; JSDT, Japanese Society for Dialysis Therapy.
